# Can body mass index influence the fracture zone in the fifth metatarsal base? A retrospective review

**DOI:** 10.1186/s13047-020-0374-6

**Published:** 2020-02-22

**Authors:** M. Pugliese, D. De Meo, E. Sinno, V. Pambianco, A. U. Cavallo, P. Persiani, C. Villani

**Affiliations:** 1 Department of Anatomical, Histological, Forensic Medicine and Orthopaedic Science, Sapienza University of Rome, Piazzale A. Moro 3, 00155 Rome, Italy; 2 Department of Orthopaedics and Traumatology, Policlinico Umberto I, Rome, Italy; 3 Division of Diagnostic and Interventional Radiology, University Hospital Policlinico “Tor Vergata”, Rome, Italy

## Background

Fifth metatarsal base fracture are common in routine orthopaedic practice [1–6]. Lawrence and Botte [7] proposed a classification based upon the position of the fracture line (zone 1: tuberosity, zone 2: meta-diaphyseal junction, zone 3: proximal diaphysis). Pathomechanically, injury patterns develop in different ways: in zone 1, a traction injury caused by peroneus brevis tendon and the lateral band of the plantar fascia determine an avulsion fracture of the tuberosity, also called “pseudo-Jones’ “fracture; in zone 2, forced foot adduction and excessive plantar flexion determine a fracture in the metaphyseal-diaphyseal junction, also called Jones’ fracture [8, 9]; in zone 3, acute over-bearing onto the area or chronic overload determine a fracture in the proximal portion of the diaphysis, distal to the intermetatarsal joint [10, 11].

To the best of the Authors’ knowledge, no study has been published to date on the relationship between the value of Body Mass Index (BMI) and the prevalence of fractures in a specific portion of the fifth metatarsal base. The aim of this study was to define the impact of BMI on fifth metatarsal base fractures location according to Lawrence and Botte classification [7].

## Methods

**Fig. 1:**
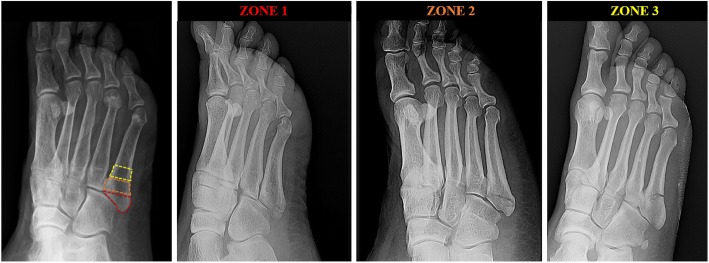
Radiographic classification of the fractures according to Lawrence and Botte^7^. The three different zones are based on anatomic landmarks (far left): proximal to the meta-diaphyseal junction involving the tuberosity (zone 1, centre left), between the lines of the intermetatarsal joint between fourth and fifth metatarsal bones involving the meta-diaphyseal junction and the intermetatarsal articular facet (zone 2, centre right), distal to the meta-diaphyseal junction involving the proximal diaphysis (zone 3, far right)

A retrospective observational analysis was performed. Patients diagnosed with fifth metatarsal base fractures between March 2016 to December 2018 were selected. Inclusion criteria were: age at presentation between 18 to 85 years-old and a twisting-type injury as a causative mechanism. Patients with additional fractures involving the foot and/or ankle, forefoot and/or hindfoot deformity, connective-tissue and/or rheumatic diseases, primary tumor or secondary localization, state of pregnancy were excluded. Electronic medical records were searched for sex, age, height, weight, mechanism of injury. Plain radiograph study (Anteroposterior, Oblique and Lateral view) were obtained and classified according to Lawrence and Botte classification (Fig. 1). Patients were classified based on BMI: < 20 kg/m^2^: underweight, between 20 and 24.9 kg/m^2^: normal weight, between 25.0 and 29.9 kg/m^2^: overweight, 30 kg/m^2^ and above: obese [6]. Statistical analysis was conducted using R V 3.4.4 (R Core Team (2018). R: A language and environment for statistical computing. R Foundation for Statistical Computing, Vienna, Austria [12]. Continuous variables were reported as mean ± standard deviation. Repeated measures design Analysis of Variance (ANOVA) and Tukey’s Honest Significant Difference test were used to compare BMI across groups. ANOVA was finally used to investigate the presence of statistically significant differences attributable to age, and Chi squared test was used to investigate the presence of statistically significant differences attributable to sex.

## Results

**Fig. 2:**
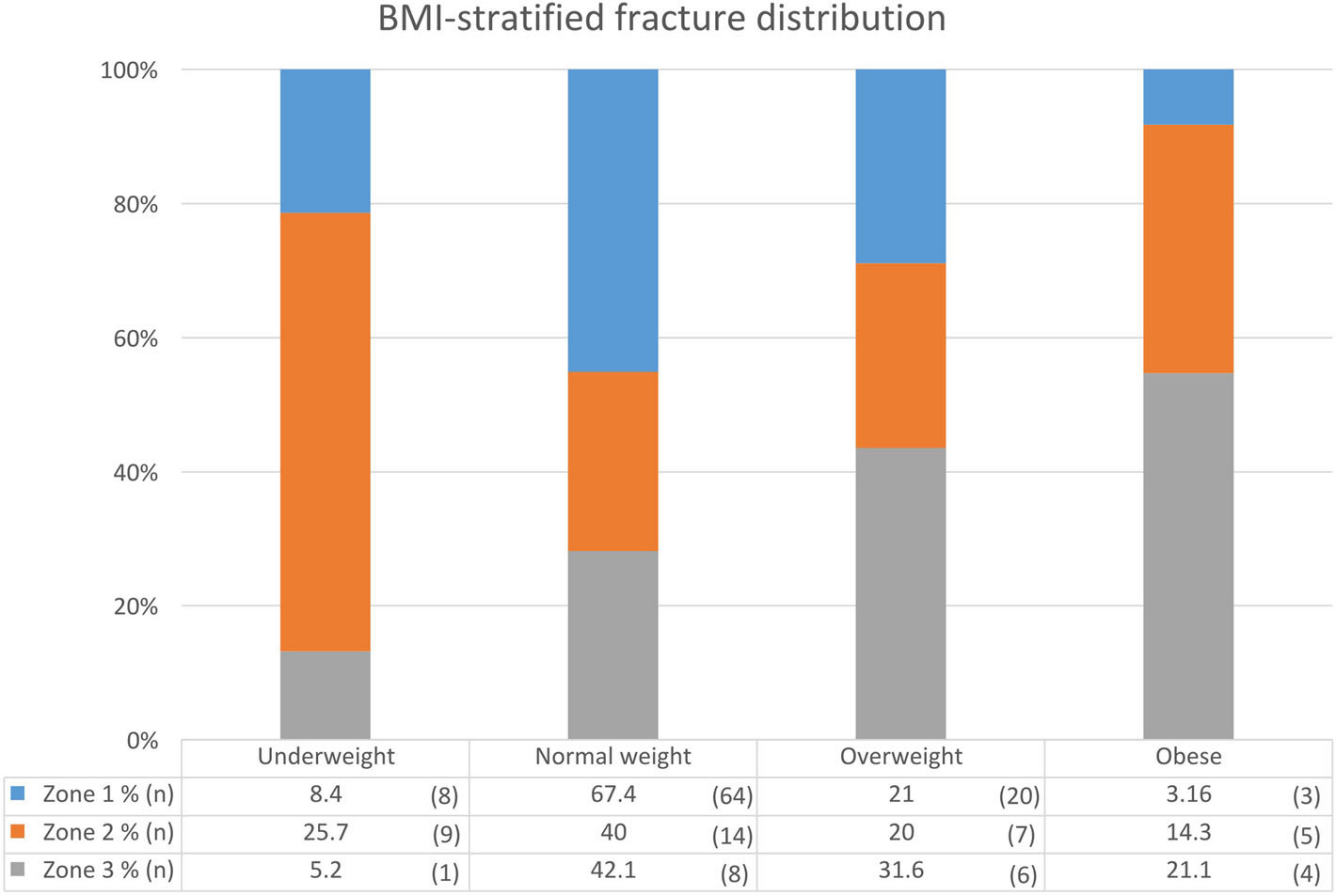
BMI-stratified fracture distribution. Data in the histogram represent the ratio between fracture zone over the total of a weight class ($\frac{\mathrm{N}\ \mathrm{patients}\ \mathrm{with}\ \mathrm{fracture}\ \mathrm{in}\ \mathrm{zone}\ \mathrm{n}}{\mathrm{Total}\ \mathrm{patients}\ \mathrm{in}\ \mathrm{considered}\ \mathrm{weight}\ \mathrm{class}}$). Data in the table represent the ratio between weight class over the total of fractures zone $\left(\frac{\mathrm{N}\ \mathrm{patients}\ \mathrm{in}\ \mathrm{considered}\ \mathrm{weight}\ \mathrm{class}}{\mathrm{Total}\ \mathrm{patients}\ \mathrm{with}\ \mathrm{fracture}\ \mathrm{in}\ \mathrm{zone}\ \mathrm{n}}\right)$. Note the growing distribution of proportion of zone 3 fractures with growing BMI (gray-coloured area)

One hundred forty-nine patients were included in the analysis. 109 (73.1%) patients were female, 40 were male. Mean age was 51.9, with a Standard Deviation (SD) of17.2 years. Mean BMI was 24 (SD = 3.8) kg/m^2^ (Table 1). According to Lawrence and Botte classification [7], 95 patients (63.8%) suffered a fracture involving Zone 1, 35 (23.4%) involving Zone 2 and 19 (12.8%) involving Zone 3. Data distribution based on BMI and zone of fracture are shown in Fig. 2. No statistically significant differences attributable to sex (*p* = 0.774) between different zones of fracture. ANOVA analysis found no statistically significant differences attributable to age (*p* = 0.379) between different zones of fracture. ANOVA analysis and post hoc Tukey test, found BMI to be significantly higher in the zone 3 (26.1, SD = 4.7 kg/m^2^) fracture group than in zone 1 (23.7, SD = 2.9 kg/m^2^) (*p* = 0.031).

**Table 1:** Demographic data of study population and divided according to Lawrence and Botte Classification

	Total	Zone 1	Zone 2	Zone 3	*p*-value
N (%)	149	95 (63.8)	35 (23.5)	19 (12.7)	–
Age (mean ± SD)	51.9 ± 17,2	53.4 ± 16.4	49.9 ± 18.4	48.4 ± 18.3	0.379^b^
Female n (%)	109 (73,1)	69 (72.6)	27 (77,1)	13 (68.4)	0.774^a^
BMI (mean ± SD)	24 ± 3.8	23.7 ± 2.9	23.7 ± 4.8	26.1 ± 4.7	0.031^b^

^a^chi-square test, ^b^ANOVA test

## Discussion

The most important finding of this study is the relationship between the prevalence of specific patterns of fifth metatarsal base fractures and BMI. The relative prevalence of fracture zones in this study reflects a trend which was already evident in the literature [6, 13]. The role of some demographic features (i.e. age, sex etc.) was investigated in its association with fifth metatarsal base fracture. While Kane et al. [6] found a statistically significant correlation between female sex and zone 1 fracture (*p* < 0.001), no such result attributable to age (*p* = 0.379) or sex (*p* = 0.774) was evident in this study sample despite the similar proportions of patients for each variable considered. In their sample, patients were stratified according to etiology into five categories: “twisting”, “fall”, “crush”, “indirect trauma”, “unknown”; the two most represented categories were “twisting” (57.2%) and “fall” (22.1%). Patients whose mechanism of injury was clearly identifiable as “twisting” were exclusively included in the study: this was done by directly questioning each patient and explicitly asking if they “fell from any kind of height”. This choice was dictated by the difficulty in discerning two “different but similar” pathomechanical categories (“twisting” and “fall”) and was done in order to minimize bias. In both studies, patients were stratified according to BMI using the same threshold values. The same boundaries were used in order to facilitate comparative analysis with the only published study which categorizes its population of fifth metatarsal base fracture patients according to BMI.

In this study a statistical analysis of data regarding BMI was performed, which showed a statistically significant difference between increasing BMI and increasing prevalence of zone 3 fractures. Despite the smaller sample, its results confirm a trend which is already visible in the analysis performed by Kane: a rising trend of obesity can be seen as the fracture moves more distally on the fifth metatarsal base. The same trend is not obvious here, but a statistically significant difference is evident. In future research, statistical analysis in larger samples can provide enough power to either confirm or disprove this trend and determine if a statistical correlation is present.

The relationship between BMI and fractures was investigated in the recent literature. It was traditionally thought that higher BMI in overweight and obese patients correlated with a lowered fracture risk due to increased Bone Mineral Density (BMD) [14–16]. On the other hand, the protective effect of higher BMI through increased BMD displays a ceiling-effect [17] and is possibly counteracted by metabolic and systemic proinflammatory effects [18], questioning several aspects of said protective factor. Therefore, even if present, it might not be able to compensate for the stronger vectors of forces at play in obese individuals [19]. Court-Brown et al. [20] investigated the relationship between fractures and obesity in the general population: no association was found between metatarsal fractures and BMI. However, neither the ordinal number nor the fractured area of the metatarsal bone were specified. In fact, the Authors are not aware of any other published study who subclassified and analysed proximal fifth metatarsal fractures in relation to BMI Since no established protective effect attributable to increased BMD is known to take place in the fifth metatarsal, the results of this study could be partially explained by acute excessive bearing onto the foot [10], further exacerbated by excess weight.

Three factors can contribute to muscle and tendon degeneration in the patient with a higher-than-normal percentage of body fat: peroneus brevis muscle dynapenia, microvasculopathy, systemic and localized chronic low-grade inflammation. Muscle mass infiltration by excess fat can lead to a combination of “sarcopenic” [21] and “dynapenic” [22] obesity, which in turn leads to a reduction of strength in the affected muscles. Also, dynapenia can contribute to poorer muscle control while falling [16], which could lead to a lack of defensive contraction of the peroneus brevis. Pathomechanically, overabundant adipose tissue can influence fracture in the overweight/obese patient by acting both on bone and on soft tissue: it is possible that not only excess weight alters the way the bone responds to weight-bearing, but also that proinflammatory factors associated to systemic dysmetabolic disorders trigger tendon degeneration [23]. The higher rate of formation of advanced glycation end-products increases the number of stable covalent cross-links within collagen fibres, which in turn alter their structure and functionality. Finally, systemic (adipokine-mediated) and localized (metalloproteinase-mediated) chronic low-grade inflammation can cause tendon damage in the long term. While it might be difficult to disentangle the impact of each factor, a combination of these might explain the lower incidence of obese patient in the zone 1/2 fracture group fractures compared to zone 3 in this study sample.

The results of this study might also have implications regarding treatment and prognosis. High BMI might have an impact in zone 3 fractures non-union, which are known to have an intrinsic risk of non-union per se [24, 25]. In fact, high BMI is a known factor for fracture non-union in the general population [26]. As current literature suggest operative treatment of zone 3 fractures either in the athlete [11, 27], in case of significant displacement [28] or established non-union [29], the treating orthopaedic surgeon might choose to warn these patients of their increased chance of non-union and suggest modifications to the treatment plan accordingly.

This study has several limitations: firstly, the sample size is small, especially when compared to other series published in the literature. Secondly, data on weight and height was gathered retrospectively through inquiry and self-report. Thirdly, despite the conscious effort to include only fractures which were a result of a “twisting-type” motion, the retrospective fashion of the study did not allow for a more thorough analysis of the injury mechanism. Lastly, results were not adjusted according to BMD or any underlying metabolic disorders: as the behaviour of BMD in the fifth metatarsal was not studied in literature or investigated by us, this aspect was not included it in the analysis.

## Conclusions

The results of this study show that BMI might play a role in the prevalence of specific fracture patterns in the fifth metatarsal base: overweight and obese patients are most numerous in the zone 3 fracture group and the risk of incurring in such fracture increases with higher BMI values. A possible explanation can be found in the secondary biomechanical and metabolic effects of excessive adipose tissue. Despite the several limitations, this study can lay ground for future research in this field: statistical analysis on larger sample size can confirm or disprove these findings, with further implications on treatment and prognosis.

## Data Availability

Please contact author for data requests.

## Abbreviations

BMD: Bone Mineral Density
BMI: Body Mass Index
SD: Standard Deviation
